# Genome-Wide Profiling of Alternative Splicing and Gene Fusion during Rice Black-Streaked Dwarf Virus Stress in Maize (*Zea mays* L.)

**DOI:** 10.3390/genes13030456

**Published:** 2022-03-02

**Authors:** Yu Zhou, Qing Lu, Jiayue Zhang, Simeng Zhang, Jianfeng Weng, Hong Di, Lin Zhang, Xin Li, Yuhang Liang, Ling Dong, Xing Zeng, Xianjun Liu, Pei Guo, Huilan Zhang, Xinhai Li, Zhenhua Wang

**Affiliations:** 1 Key Laboratory of Germplasm Enhancement, Physiology and Ecology of Food Crops in Cold Region, Department of Agriculture, Northeast Agricultural University, Harbin 150030, China; zhouyu0924@126.com (Y.Z.); luqingtx0803@163.com (Q.L.); 15692360557@163.com (J.Z.); zsm07249921@163.com (S.Z.); dihongdh@163.com (H.D.); neauzla@163.com (L.Z.); lixin11170104@163.com (X.L.); liangyuhang19986@163.com (Y.L.); dongling_1980@163.com (L.D.); zengxing980@hotmail.com (X.Z.); iflyu@163.com (X.L.); 18847163925@163.com (P.G.); z1025231075@163.com (H.Z.); 2 Institute of Crop Science, Chinese Academy of Agricultural Sciences, Zhongguancun South Street, Haidian District, Beijing 100081, China; wengjianfeng@caas.cn

**Keywords:** rough dwarf disease, alternative splicing, fusion gene, *Zea mays* L., differentially expressed genes, virus stress

## Abstract

Rice black-streaked dwarf virus (RBSDV) causes maize rough dwarf disease (MRDD), which is a viral disease that significantly affects maize yields worldwide. Plants tolerate stress through transcriptional reprogramming at the alternative splicing (AS), transcriptional, and fusion gene (FG) levels. However, it is unclear whether and how AS and FG interfere with transcriptional reprogramming in MRDD. In this study, we performed global profiling of AS and FG on maize response to RBSDV and compared it with transcriptional changes. There are approximately 1.43 to 2.25 AS events per gene in maize infected with RBSDV. *GRMZM2G438622* was only detected in four AS modes (A3SS, A5SS, RI, and SE), whereas *GRMZM2G059392* showed downregulated expression and four AS events. A total of 106 and 176 FGs were detected at two time points, respectively, including six differentially expressed genes and five differentially spliced genes. The gene *GRMZM2G076798* was the only FG that occurred at two time points and was involved in two FG events. Among these, 104 GOs were enriched, indicating that nodulin-, disease resistance-, and chloroplastic-related genes respond to RBSDV stress in maize. These results provide new insights into the mechanisms underlying post-transcriptional and transcriptional regulation of maize response to RBSDV stress.

## 1. Introduction

Maize rough dwarf disease (MRDD) is caused by rice black-streaked dwarf virus (RBSDV) in regions of China where maize is grown in the summer months [1]. It is a viral disease that occurs around the world [2,3,4]. In China, maize, rice, and wheat are all susceptible to RBSDV [5], which belongs to the family *Reoviridae* and the genus *Fijivirus* [6]. RBSDV is spread by the brown planthopper (SBPH, *Laodelphax striatellus*) [7]. MRDD can inflict severe yield losses in maize, ranging from 30% to 100%. In China, cases are particularly acute in the Huai and Yellow River valleys [8,9].

To control MRDD, significant research into its resistance mechanism and pathogenesis has been performed. The primary QTL responsible for RBSDV resistance in maize was identified on bin 8.03 based on fine mapping [10,11]. There were 10 annotated candidate genes responsible for MRDD resistance that were annotated with antifreeze proteins, ethylene-responsive transcription factors, and phosphatidylinositol kinase and were identified during a genome-wide association study (GWAS) [12]. The resistance gene Rab GDP dissociation inhibitor α (*RabGDIα*) in maize is a susceptibility factor for RBSDV in which the alternative splicing of exon-10 was not banded with the RBSDV P7-1 protein, leading to quantitative recessive resistance to MRDD [13]. Profiling of a maize proteome during the flowering stage demonstrated that glycolysis and starch metabolism, followed by morphology and development, were responses to RBSDV [14]. Dual transcriptome analysis of the MRDD plant demonstrates that the chloroplast, cell wall, and development-related pathways and genes were all significantly altered when responding to an RBSDV infection [15,16]. *miRNA8155* and *miR169i-p5*, along with their target genes, *GRMZM2G031169* and *GRMZM2G069316*, have been found to respond to RBSDV infections in maize with the annotation functions of the nucleole and an NAD(P)-binding Rossmann-fold protein [15].

Alternative splicing (AS), also known as differential splicing, refers to pre-mRNA processing events. These events are regulated during gene expression and produce a single gene that codes several proteins, including the skipping exon (SE), alternative 3′ splicing site (A3SS), intron retention (RI), alternative 5′ splicing site (A5SS), and mutually exclusive exons (MXE) [17,18]. AS typically occurs in eukaryotes, which significantly increase the protein biodiversity that the genome can encode [18]. For instance, approximately 95% of multiexonic genes exist with AS [19]. The structures, functions, and subcellular locations of these proteins can be altered by AS transcripts [20,21,22]. AS plays a significant role in post-transcriptional responses to biotic and abiotic stresses in plants [23,24], including development in maize endosperm [25], the evolution of maize or soybean [26,27], drought and heat in maize or wheat [22,28,29], and plant-virus interactions in *Brachypodium distachyon* [30] and wheat [31]. However, there are no reports about whether AS plays a role in maize response to RBSDV.

Gene fusion (GF) or fission events occur when chimeric genes composed of two or more genes with connected end-to-end coding regions are controlled by the same regulatory sequence. These chromosomal rearrangements are triggered by DNA double-strand breakage [32], including chromosomal translocation, deletion, and inversion. Gene fusion is closely related to human diseases, particularly cancer. GF events account for approximately 0.5% of all prokaryotic genes, whereas most gene fusions that have been characterized occur in fungi and bacteria. Several studies have found gene fusions during microbial metabolism [33]. GF events have produced several protein chimeras in microbes, plants, and other organisms. GF and fission events occurring in eukaryotes are more complicated than those that occur in prokaryotes, so their contributions to genomic evolution are still poorly understood [34]. Therefore, gene fusion or fission has contributed to the significant diversity of terpene synthases that occur in plants [35]. The majority of GF events in plants have been identified with traditional biochemical techniques and functional genomics using existing databases of transcriptomes, particularly in maize [34,36]. However, it remains unclear whether GF participates in transcriptional reprogramming during maize-RBSDV interactions.

In this study, we compared transcriptional changes and performed global profiling of AS and GF to assess the response of maize to RBSDV. We also outlined the AS and FG landscape across the whole maize genome to examine its response to RBSDV. Our findings demonstrate that maize genomes have different FG and AS responses to viral infections and that differentially expressed genes (DEGs) can help regulate the FG and AS events that respond to RBSDV, as opposed to independently responding to the virus.

## 2. Materials and Methods

### 2.1. Plant Materials, Artificial Inoculation of RBSDV, and Sequencing of RNA

We used RNA-seq data from our previous study (NCBI SRA database: PRJNA299369; SRR numbers: SRR2758150 to SRR2758159, SRR2758161 to SRR2758170, and SRR2758174 to SRR2758177) according to the process outlined below [15]. The maize inbred line B73, which is highly susceptible to RBSDV, was artificially inoculated with RBSDV at the V3 stage with SBPH of 1% viruliferous. This took place in Nanjing, Jiangsu Province, China. B73 seedlings were artificially inoculated at the V3 stage, using SBPH and with no virus as the control. Three technical replicates were performed for all treatments. The leaves were sampled 1.5 and 3.0 DAI (days after inoculation). The artificial inoculation was verified using qRT-PCR at the V3a (1.5 d), V3b (3.0 d), V6, V9, and V12 stages of B73 plants. All seedlings inoculated with viruliferous SBPH or virus-free SBPH were transplanted to the field for growth inside insect-proof netting. MRDD symptoms/number of B73 plants were analyzed at the VT (tasseling) stage.

TRIzol^®^ reagent (Invitrogen, CA, USA) was used to extract the total RNA from all samples, according to the manufacturer’s instructions. An RNA 6000 Nano LabChip kit (Agilent, CA, USA) with RIN number >7.0 and a Bioanalyzer 2100 (Agilent, CA, USA) were used to assess the purity and concentration of the RNA. RNA sequencing was performed using an Illumina HiSeq 2500 (Hangzhou, China) on 12 transcriptome libraries, including three biological replicates, and each was inoculated with the RBSDV virus or the control. For every sample, five seedings were combined per biological replicate. More than 4G of data of each transcriptome library were assembled using Trinity (https://www.trinitytec.net/ accessed on 12 January 2022) [15].

### 2.2. Identification of RBSDV-Responsive AS Events and Gene Fusions

For the reference genome, a database of the maize genome, ZmB73RefGenv4, was used. To identify AS events that occurred during maize’s response to RBSDV, SpliceGrapher software (https://sourceforge.net/p/splicegrapher/wiki/Home/ accessed on 12 January 2022)was used to detect the transcripts [37]. In the 12 transcriptome libraries, five kinds of AS events, starting sites, and stopping sites were measured, including SE, RI, A3SS, A5SS, and MXE. The RBSDV-responsive AS events were compared between the samples of virus and control samples, with *p*-values less than 0.05. We then calculated the total alternatively spliced events and the ratio of each event and generated a Venn diagram to display the AS events at 1.5 and 3.0 DAI. We obtained each gene’s read count from the results of this mapping, which were translated to fragments per kilobase of transcript per million mapped reads (FPKM). Significant differences in the expression of the genes were considered when *p* < 0.05 and |log_2_FC| ≥ 1.

FusionMap software (http://www.arrayserver.com/wiki/index.php?title=FusionMap accessed on 12 January 2022) was used to identify gene fusion in the genome during maize response to RBSDV. The fusion gene was detected when the coding regions of two or more genes were arrayed end-to-end and in the same regulatory sequence (promoter, enhancer element, ribosomal binding sequence, terminator). Fusion gene events were detected in all three replicates and were found in both the viruliferous and control maize at 1.5 and 3.0 DAI, respectively.

### 2.3. Functional Annotation and GOs of DEGs, DSGs, and FGs

The NCBI database and the maize GDB database were used to annotate the genes. Analysis of gene ontology (GO) enrichment provided the GO terms of the DEGs, differentially spliced genes (DSGs), and fusion genes (FGs). Compared to the average values in these databases, they were all significantly enriched. Significantly enriched GO terms were located using GO Term Finder (http://www.yeast-genome.org/help/analyze/go-term-finder accessed on 12 January 2022) and a hypergeometric test. The maize genes were annotated by GO annotations, which came from the Gene Ontology Consortium (http://www.geneontology.org/ accessed on 12 January 2022).

### 2.4. Validation of RT-PCR of AS Events and qRT-PCR of DEGs

Three biological replicates and three technical replicates were used to validate differentially expressed and differentially spliced genes. Oligo-dT primers and reverse-transcriptase M-MLV were used to treat the total RNA with DNase I, according to the instructions of the manufacturer. A total of 500 ng of RNA was used from each sample, for which reverse transcription was performed with a TransScript^®^ one-step gDNA removal kit and a cDNA Synthesis SuperMix (TransGen Biotech, Beijing, China). A 10 μL reaction volume was used to perform RT-PCR, and a primer pair was designed for each gene to increase each splice variant (isoforms 1 and 2) within a reaction. An Analytik Jena real-time PCR detection system (Analytik Jena AG, Jena, Germany) was used to perform qRT-PCR; the control was the maize actin gene. Table S1 displays the primers used in the qRT-PCR and RT-PCR analyses.

## 3. Results

### 3.1. Identifying Alternative Splicing Events in Maize

To address whether early AS events are involved in maize response to RBSDV, we first performed RNA-seq analysis using maize seedlings at 1.5 and 3 DAI upon RBSDV infection. Previous studies have demonstrated that there are thousands of DEGs playing various roles in RBSDV stress response. This study used the same RNA-seq data to further analyze the AS profile of maize infected with RBSDV (Table S1).

We identified 5824 and 6248 AS events in 4066 and 2782 maize genes at 1.5 or 3.0 DAI, respectively. This is approximately 1.43 to 2.25 AS events per gene, which accounts for 8.69% to 12.70% of all expressed maize genes, respectively. A total of 506 AS events from 383 genes were detected at both 1.5 and 3.0 DAI after RBSDV infection.

**Figure 1:**
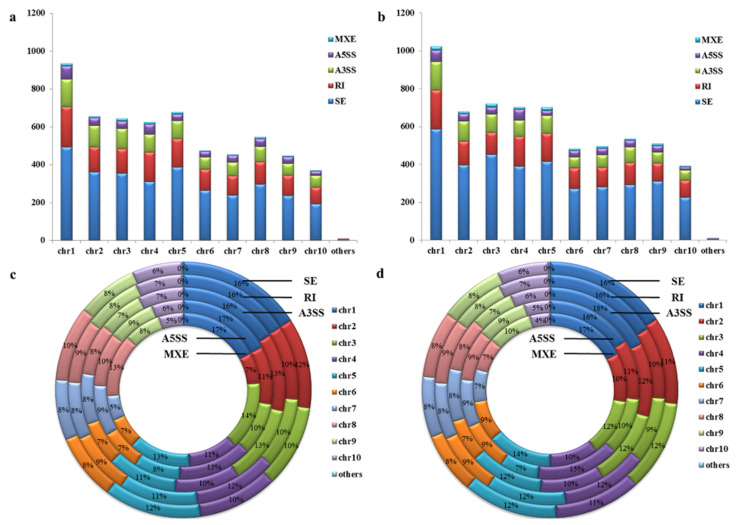
Overview of alternative splicing events in maize infected with RBSDV. (**a**) Distribution of various AS event modes from chromosome 1 to 10 in maize infected with RBSDV at 1.5 DAI; (**b**) distribution of various AS event modes from chromosome 1 to 10 in maize infected with RBSDV at 3.0 DAI; (**c**) distribution of different modes of genes from chromosome 1 to 10 in maize infected with RBSDV at 1.5 DAI; (**d**) distribution of different gene modes from chromosome 1 to 10 in maize infected with RBSDV at 3.0 DAI.

In 10 chromosomes of maize, AS events ranged from 369 to 933 detected at 1.5 DAI (Figure 1a) and 392 to 1022 detected at 3.0 DAI (Figure 1b). At 1.5 DAI, there were comparable distributions of AS types in 10 chromosomes of maize; the most common were SE events (49.76–56.87%), RI events (19.94–24.72%), A3SS events (13.50–17.40%), A5SS events (5.17–8.83%), and MXE events (1.05–2.18%) (Figure 1a). A similar trend was observed at 3.0 DAI; the most common were SE events (54.49%–62.64), followed by RI events (16.25–23.21%), A3SS events (11.57–15.76%), A5SS events (3.85–8.12%), and MXE events (1.50–2.56%) (Figure 1b). Different kinds of genes were detected in chromosome 1 (16.00–18.00%), followed by chromosome 4 (10.00–15.00%), and chromosome 3 (9.00–14.00%) at both 1.5 and 3.0 DAI (Figure 1c,d).

### 3.2. Identifying RBSDV Stress-Responsive AS Events in Maize

AS events that respond to RBSDV stress splice the isoforms of a gene experiencing changes in its differential expression following stress events. In the present study, stress-responsive AS events were defined as having a *p*-value less than 0.05. Ultimately, 1119 stress-responsive AS events related to 985 genes were identified at 1.5 DAI, whereas 1113 stress-responsive AS events correlated with 975 genes were identified at 3.0 DAI.

**Figure 2:**
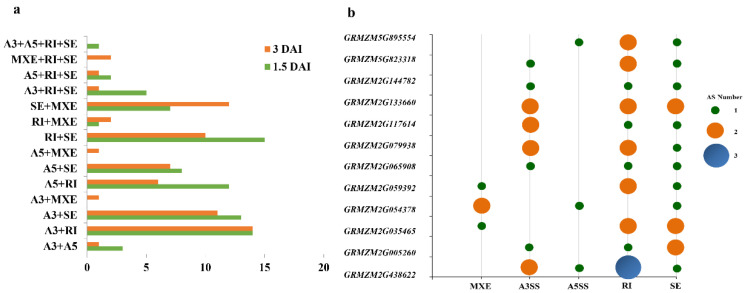
Comparison analysis of AS events in maize response to RBSDV. (**a**) Distribution of different AS modes of genes in maize infected with RBSDV at 1.5 and 3.0 DAI. (**b**) Genes with different AS events.

We found that 506 AS events from 383 genes were detected at both 1.5 and 3.0 DAI, including 231 RI, 182 SE, 121 A3SS, 48 A5SS, and 18 MEX events with 199, 140, 106, 45, and 16 genes, respectively. Some of these AS genes were spliced at the same site at 1.5 and 3.0 DAI, including 153 RI, 74 SE, 74 A3SS, 23 A5SS, and 4 MEX events (Figure 2a,b). In the 383 genes with 506 AS events, 233 genes had one AS mode, and 138 genes had two AS modes. Of these, there were 73 AS events at 1.5 DAI and 65 AS events at 3.0 DAI. Most modes were genes with both A3SS and RI from 28 genes. Eleven genes have three AS events, including six genes with A3SS, RI, and SE events (*GRMZM2G144782*, *GRMZM2G005260*, *GRMZM2G065908*, *GRMZM2G079938*, *GRMZM2G117614*, and *GRMZM5G823318*), three genes with A5SS, RI, and SE events (*GRMZM2G054378*, *GRMZM2G133660*, *GRMZM5G895554*), and two genes with MEX, RI, and SE events (*GRMZM2G035465* and *GRMZM2G059392*). *GRMZM2G438622* was only detected in the four AS modes (A3SS, A5SS, RI, and SE) (Table S2).

### 3.3. Comparing Differentially Spliced Genes and Differentially Expressed Genes

**Figure 3:**
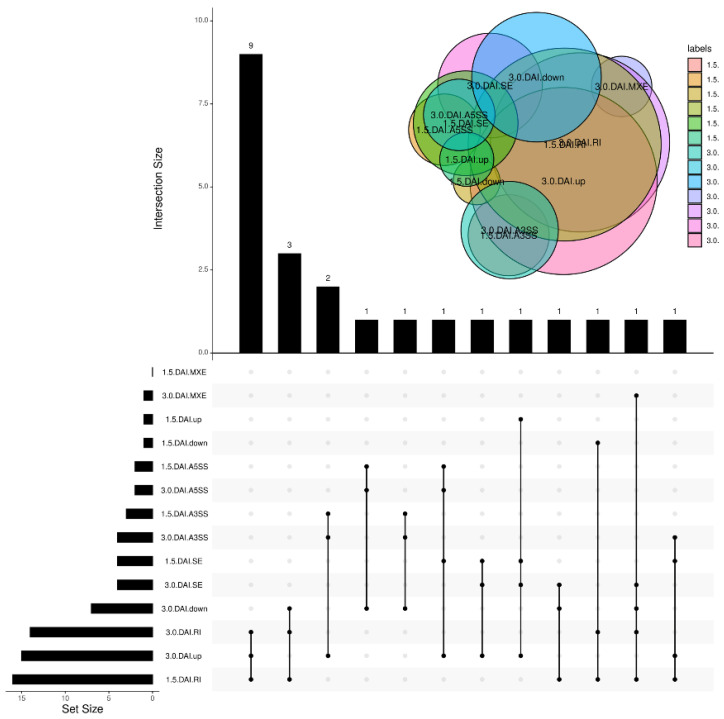
Analysis of differentially spliced genes and differentially expressed genes.

To assess the association between DEGs and AS, genes altering their transcription and AS in response to RBSDV were compared. A total of 23 DSGs with two or more AS events displayed patterns of differential expression (DEGs: Figure 3 and Table S3), including 30 RI, 8 SE, 7 A3SS, 4 A5SS, and 1 MXE event. Two DSGs (8% of the total) exhibited differential expression patterns at 1.5 DAI, whereas 22 DSGs exhibited differential expression patterns at 3.0 DAI.

Among these 23 DSGs, 53 AS events were detected, including six genes on chromosome 1, three genes on chromosome 2, three genes on chromosome 3, two genes on chromosome 4, two genes on chromosome 5, one gene on chromosome 7, four genes on chromosome 9, and two genes on chromosome 10 (Table S3). The most common were the nine genes combined with RI events and upregulated expression: *GRMZM2G004207* (*p* < 0.01), *GRMZM2G011523*, *GRMZM2G028307*, *GRMZM2G037452* (*p* < 0.05), *GRMZM2G047456*, *GRMZM2G102183*, *GRMZM2G117989*, *GRMZM2G354909*, and *GRMZM5G838098*. Three genes, *GRMZM2G028325*, *GRMZM2G058447*, and *GRMZM2G376416*, combined with RI events and downregulated their expression. Two genes, *GRMZM2G113512* and *GRMZM2G412986*, combined with A3SS events at 1.5 and 3.0 DAI and upregulated their expression. Five genes were detected in two AS events and regulated their expression differently: *GRMZM2G451716* (SE at 1.5 and 3.0 DAI, which upregulated expression), *GRMZM2G152105* (A3SS event at 1.5 and 3.0 DAI, which downregulated expression), *GRMZM2G065214* (A5SS event at 1.5 and 3.0 DAI, which downregulated expression), *GRMZM2G060837* (RI at 1.5 and 3.0 DAI, which downregulated expression, *p* < 0.01), *GRMZM2G043493* (RI at 1.5 DAI and SE at 3.0 DAI, which downregulated expression). Two genes were detected in three AS events and upregulated their expression: *GRMZM2G144782* (RI and SE at 1.5 DAI and A3SS at 3.0 DAI), *GRMZM2G008607* (A5SS at 1.5 and 3.0 DAI and SE at 1.5 DAI). *GRMZM2G029912* (*p* < 0.01) was the only DSG with SE events, and its expression was upregulated at two time points. *GRMZM2G059392* showed downregulated expression and four AS events, which contained RI at 1.5 and 3.0 DAI and MXE and SE at 3.0 DAI.

The 13 genes of these 23 DSGs are involved in annotation, including six encoded proteins. This includes the CHY-type/CTCHY-type/RING-type Zinc finger protein (*GRMZM2G144782*), gl1 protein (*GRMZM2G029912*), NOD26-like membrane integral protein ZmNIP2-1 (*GRMZM2G028325*), protein MEI2-like 1 (*GRMZM2G451716*), putative protein kinase superfamily protein (*GRMZM2G113512*), and putative xyloglucan endotransglucosylase/hydrolase protein 30 (*GRMZM2G060837*). Seven other annotation genes were related to the enzyme, including (+)-neomenthol dehydrogenase (*GRMZM2G354909*), barwin-like protein (*GRMZM2G117989*), Bowman-Birk type wound-induced proteinase inhibitor WIP1 (*GRMZM2G011523*), carboxy-lyase (*GRMZM2G059392*), peroxidase 73 (*GRMZM2G047456*) serine/threonine-protein kinase (*GRMZM2G004207*), and malate synthase 1 (*GRMZM2G102183*).

### 3.4. Validation of Differentially Expressed and Differentially Spliced Genes

**Figure 4:**
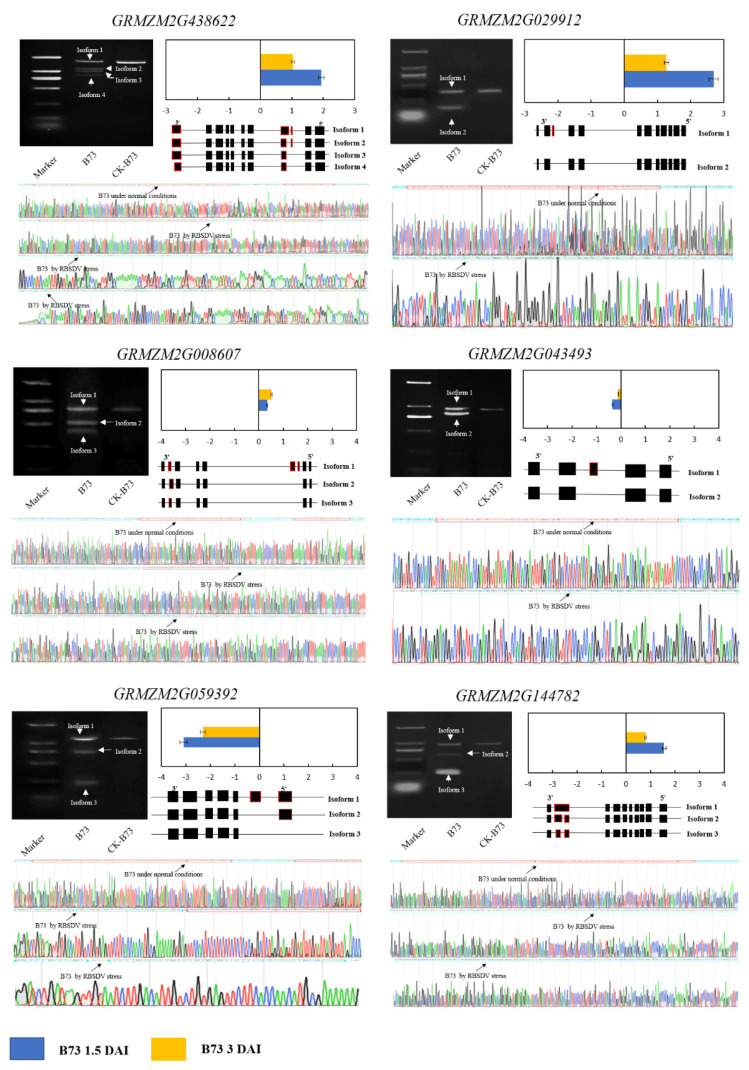
Experimental validation of AS events, sequencing validation, and expression levels in maize response to RBSDV.

To confirm the stress-responsive AS events identified by RNA sequencing, six candidate genes that exhibited significant differential expression levels were chosen for additional qRT-PCR and sequencing analysis: *GRMZM2G438622*, *GRMZM2G029912*, *GRMZM2G008607*, *GRMZM2G043493*, *GRMZM2G059392,* and *GRMZM2G144782* (Figure 4 and Table S5). Analysis of the qRT-PCR results and RNA-seq data demonstrated consistent expression trends for all six genes. Four genes (*GRMZM2G438622*, *GRMZM2G029912*, *GRMZM2G008607*, and *GRMZM2G144782*) showed upregulated expression, and two genes (*GRMZM2G043493* and *GRMZM2G059392*) showed downregulated expression when exposed to RBSDV stress. Two genes (*GRMZM2G438622* and *GRMZM2G144782*) displayed significantly higher expression levels at 1.5 DAI than at 3.0 DAI (*p* < 0.05), one gene (*GRMZM2G008607*) displayed significantly higher expression levels at 3.0 DAI than at 1.5 DAI (*p* < 0.05), and two genes (*GRMZM2G043493* and *GRMZM2G059392*) displayed significantly lower expression levels at 1.5 DAI than at 3.0 DAI (*p* < 0.05). One candidate gene, *GRMZM2G029912*, showed significantly upregulated expression at 1.5 DAI (*p* < 0.05) and significantly downregulated expression at 3.0 DAI (*p* < 0.05).

Conserved primer pairs were used for the amplification of a single reaction. Six DSGs (*GRMZM2G438622*, *GRMZM2G029912*, *GRMZM2G008607*, *GRMZM2G043493*, *GRMZM2G059392*, and *GRMZM2G144782*) showed consistent AS patterns, and their profiles were revealed by RNA-seq data. Based on RNA sequencing, isoform 1 of *GRMZM2G029912* and *GRMZM2G043493* was only expressed under normal conditions, whereas isoform 2 was dramatically induced by RBSDV stress. Three genes (*GRMZM2G008607*, *GRMZM2G059392* and *GRMZM2G144782*) expressed isoform 1, isoform 2, and isoform 3 under RBSDV stress. *GRMZM2G438622* only expressed isoform 1 under normal conditions, whereas isoform 2, isoform 3, and isoform 4 were dramatically induced by RBSDV stress.

### 3.5. Comparative Analysis of Fusion Genes under RBSDV Stress

Genes subject to fusion and transcriptional changes were compared to identify FGs involved in maize response to RBSDV. A total of 53 and 88 GF events in maize were detected at 1.5 and 3.0 DAI, respectively. The most common splice pattern was GT-AG, with 66 and 34 at 1.5 and 3.0 DAI, respectively. Nine overlap FG events were detected at both time points: *GRMZM2G404702*-*GRMZM2G151406*, *GRMZM2G069203*-*GRMZM5G816110*, *GRMZM2G01933*5-*GRMZM2G095299*, *GRMZM2G169326-GRMZM2G561273*, *AC196489.3_FG002*-*GRMZM2G556667*, *GRMZM2G076798*-*GRMZM2G008649*, *GRMZM2G014917*-*GRMZM2G023694*, *GRMZM2G424595*-*GRMZM2G090100,* and *GRMZM2G065298*-*GRMZM2G065066*. A total of 19 genes were identified in the FG events at 1.5 and 3.0 DAI, with annotations of cation-transporting ATPase HMA5, serine acetyltransferase 1, and ubiquitin carboxyl-terminal hydrolase 2. In this study, *GRMZM2G076798* was the only FG that occurred at two time points and two related FG events: *GRMZM2G076798*-*GRMZM2G008649* at 1.5 and 3.0 DAI and *GRMZM2G076798*-*GRMZM5G812860* at 1.5 DAI.

A total of 106 and 176 FGs were detected at 1.5 and 3.0 DAI, respectively. Six of these genes showed differential expression, including five upregulated expression genes (*GRMZM2G108537*, *GRMZM2G088053*, *GRMZM2G475380*, *GRMZM2G046331*, and *GRMZM2G404702*) and one downregulated expression gene (*AC217499.3_FG003*). Five FGs were detected in AS events, including three DSGs with two AS events (*GRMZM2G329040* with A3SS and SE, *GRMZM2G032376* with A3SS and RI, and *GRMZM5G871727* with A3SS and RI), and *GRMZM2G081668* and *GRMZM2G042889* with SE events.

### 3.6. Analyzing the Biological Functions of DEGs, DSGs, and FGs

**Figure 5:**
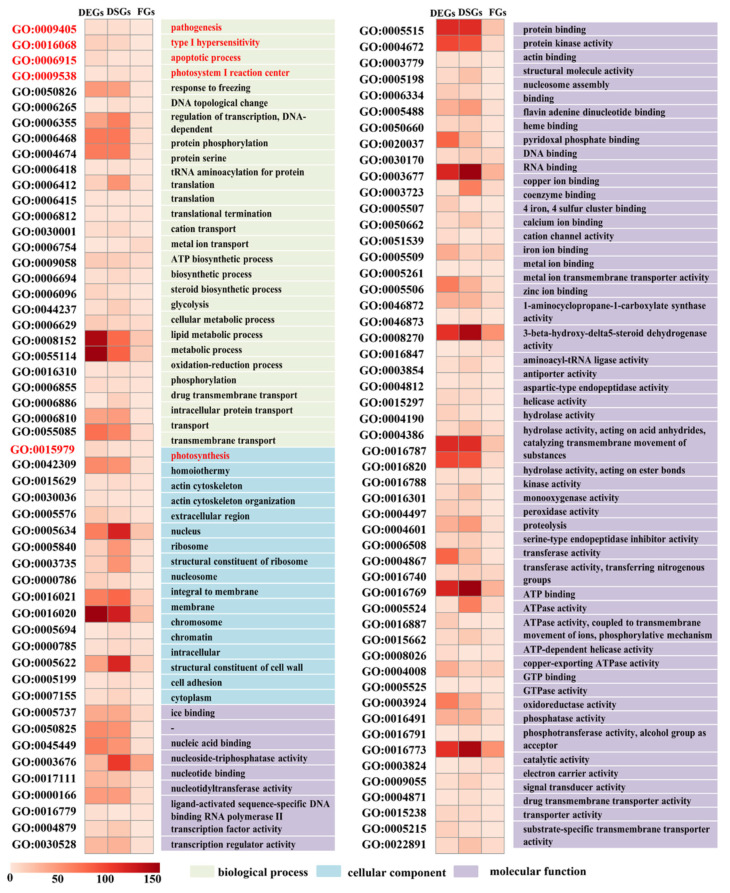
Analysis of functional enrichment of DSG&DEG&FG-overlapped genes Color scale indicates enrichment folds of various GO terms. Only significantly enriched terms (*p* < 0.05, enrichment fold ≥ 1.5) are displayed. The red colored words are related to the regulation pathway of maize rough dwarf disease resistance.

Gene ontology (GO) enrichment analysis was conducted on DSG-specific, DEG-specific, FG-specific, DSG&DEG-overlapped, and DSG&DEG&FG-overlapped genes to analyze the biological functions of genes involved in RBSDV response. A total of 459 GOs, 676 GOs, and 161 GOs were correlated with 1105 DEGs, 1539 DSEs, and 255 FGs, respectively; 325 GOs were identified in DEGs and DSGs. In DSG&DEG&FG-overlapped genes, 104 GOs exhibited enrichments involving biological processes (pathogenesis, type I hypersensitivity, apoptotic process, photosystem I reaction center, response to freezing, or metabolic processes), cellular components (photosynthesis, homoiothermy, actin cytoskeleton, nucleus, and structural constituent of cell wall), and molecular functions (ice binding, nucleic acid binding, transcription regulator activity, peroxidase activity, and metal ion transmembrane transporter activity) (Figure 5).

A total of three DEGs, six DSGs, and two FGs were identified in GO:0009405 during pathogenesis annotation. These genes were annotated as nodulin proteins (FG and DEG: *GRMZM2G108537*), protein-enhanced disease resistance 2 (DSG: *GRMZM2G160927*), nuclear pore complex protein NUP1 (DSG: *GRMZM2G329300*), and integral membrane protein-like genes (DSG: *GRMZM2G071378*). GO:0006915, which was annotated as an apoptotic process, included six DEGs, two DSGs, and one FG, with functions of Bcl-2-associated athanogene (BAG) family-related (DEG: *GRMZM2G018988*, *GRMZM2G063162*, and *GRMZM2G097135*) and disease resistance protein RPS2 (DEG: *GRMZM2G032602*). GO:0016068//type I hypersensitivity contained 11 DEGs, 11 DSGs, and two FGs, which were annotated as chloroplastic or chlorophyllic (DEG: *GRMZM2G021256*, DSG: *GRMZM2G121960*, FG: *GRMZM2G109561*), probable serine/threonine-protein kinase SIS8 (DSG and FG: *GRMZM2G127632*), and a transcription factor of MYB and WRKY (DSG: *GRMZM2G163291* and DEG: *GRMZM5G851490*). Two GOs associated with photosynthesis were also detected in DSG&DEG&FG-overlapped genes: GO:0015979//photosynthesis (including six DEGs, four DSGs, and three FGs) and GO:0009538//photosystem I reaction center (including one DEG, two DSGs, and one FG). The function of these genes was related to two photosynthesis GOs and showed photosystem-related or chloroplastic properties (DEG: *GRMZM2G024150*; DSG: *GRMZM2G112728*; FG: *GRMZM2G017290* and *GRMZM2G174984*), disease resistance protein RPS2 (DEG: *GRMZM2G032602*), and BAG family-related protein (DEG: *GRMZM2G063162* and *GRMZM2G097135*) (Table 1).

## 4. Discussion

### 4.1. AS Events Participating in Maize Response to RBSDV

Alternative splicing (AS) was first identified in calcitonin and immunoglobulin genes [25,38,39]. Previous studies detected AS in 60% of plant genes [25,40]. AS events typically include signal transduction, growth, abiotic stresses, and circadian rhythms [41,42], and AS has recently been found to play a major role in these processes during stress events, such as drought, heat, and disease [43,44,45,46,47]. In maize, AS events were identified that responded to drought [48,49], development [29], and endosperm [25]. AS events can provide a novel strategy for plant response to virus stresses. AS isoforms can be produced in plants to enhance their transcriptome plasticity and pathogen resistance, helping Brachypodium distachyon defend against the Panicum mosaic virus [30], wheat cope against powdery mildew [31], and *Arabidopsis* against *Ralstonia solanacearum* [50]. However, it has not been explored whether AS is involved in maize resistance to various viral infections, except that previous studies showed that maize resistance to MRDD is regulated by many genes that all contribute to overall resistance [10,29] and that Helitron insertion of *ZmGDIα* (*ZmGDIα*-hel) AS events was found to reduce the disease severity index by 30%, which makes it a major MRDD resistance gene [13]. An SE event of the *ZmGDIα* gene was also detected at 3.0 DAI, whereas 1119 and 1113 stress-responsive AS events associated with 985 and 975 genes were identified at 1.5 and 3.0 DAI, respectively (Figure 1). Five AS modes were explored at two time points, and the proportions of each AS mode were similar. Some DSGs showed various AS modes at 1.5 and 3.0 DAI, which could be due to the extended temporal effects of RBSDV in maize. The exosome complex component RRP45B-like protein (*ZmRRP45B*, *GRMZM2G438622*) was the only gene detected in four AS modes (A3SS, A5SS, RI, and SE) that responded to the RBSDV infection. RRP45B and RRP45A were duplicate genes of RRP45, serving particular functions during the growth and development of plants and contributing unequally to exosome activity in vivo [51,52]. RRP45B was referred to as post-transcriptional gene silencing (PTGS) with cytosolic RNA exosome to the Ski complex in *Arabidopsis* [53]. In *Arabidopsis*, other RRP-like proteins, including RRP42, play a significant role during the development of female gametophytes and mesophyll cell morphogenesis [54], and RRP41L contributes to both seed germination and early seedling growth [52]. The function of RRP-like proteins has not been reported in maize. However, in this study, *ZmRRP45B* displayed significant transcriptome plasticity in response to RBSDV.

### 4.2. Combined Analysis of DEGs and DSEs in Response to RBSDV in Maize

Changes in the gene expression of splicing factors can affect developmental processes relating to various tissues. A proportion of candidate DSGs may play important roles in plant viruses with one splicing isoform, whereas its alternative isoform was only induced under stressed conditions, which then participated in modulating responses to virus stresses in plants. This includes the development of seeds, in which the U2AF splicing factor ROUGH ENDOSPERM3 helps regulate the relationship between the endosperm and the embryo [55]. Different gene expressions related to splicing factors is a significant contributor to alterations seen in stress-induced alternative splicing [56,57,58]. For example, in *Arabidopsis*, overexpression of the splicing factor SAD1 increased splicing accuracy and salt stress tolerance. This emphasizes that mRNA changes at the transcriptional and post-transcriptional levels can provide a better understanding of stress and development. A previous study demonstrated that only 45 genes overlapped between DEGs and DSGs during the *Arabidopsis* response to R. solanacearum infection [50]. Similarly, in maize, 23 genes that overlapped between DSGs and DEGs were identified at 3.0 DAI, whereas RT-PCR was used to verify the predicted AS pattern (Table S3). *GRMZM2G029912* was the only upregulated differentially spliced gene (DSG) and was also detected during the SE event. Homologous gene function in rice is related to production, the surface of the seedling leaves, wax synthesis, and stress resistance [59,60,61]. In maize, *GRMZM2G029912* is involved in cuticular wax deposition, regulates flowering time, and aids in photosynthesis, all of which are related to classic MRDD symptoms [62,63]. *GRMZM2G059392* showed downregulated expression and four AS events, which contained RI events at 1.5 and 3.0 DAI, as well as MXE and SE events at 3.0 DAI. The encoded protein cytokinin riboside 5′-monophosphate phosphoribohydrolase LOG of *GRMZM2G059392* could be from reproductive and vegetative tissues involved in abiotic stress in maize [64].

### 4.3. Potential Role of FG in Maize-RBSDV Interaction

Gene fusion of eukaryotes is more complicated than that of prokaryotes; however, their specific role in genomic evolution remains. Gene fusion is an uncommon process that typically occurs in higher plant genomes, although this process can still be used to assess evolutionary relationships between plants and other eukaryotes. Previous studies have found that gene fusion can occur more often than fission in prokaryote genomes [65,66,67,68]. However, in eukaryote genomes, such as rice, the opposite result was observed in cDNA-supported annotations [36]. This requires further verification in additional eukaryotic genomes. There could be a significant relationship between gene fusion in humans/animals and disease. In this study, we detected gene fusion during maize response to RBSDV stress. Previous studies consistently demonstrated that gene fusion is involved in the response to these viral infections. Candidate pairs of 84 gene fusion events were identified on the same chromosome, including 18 on chromosome 1, 11 on chromosome 1, 9 on chromosome 3, 3 on chromosome 4, 13 on chromosome 5, 12 on chromosome 6, 6 on chromosome 7, 3 on chromosome 8, 5 on chromosome 5, and 4 on chromosome 10. However, 57 other gene fusion events contained candidate pairs in different chromosomes, whereas two FGs in *GRMZM2G379053*-*GRMZM6G810942* were detected on chromosome 6 and scaffold_89 (Table S4).

Due to the temporal effect of the extension of RBSDV in maize, some GF events were only found at one time point. Nevertheless, some results were consistently detected, such as 10 GF events that simultaneously occurred at 1.5 and 3.0 DAI. The annotation function of some candidate pairs was similar to that of the same gene fusion events, including cation-transporting ATPase HMA5 of *GRMZM2G404702*-*GRMZM2G151406*, ubiquitin carboxyl-terminal hydrolase 2 of *GRMZM2G014917*-*GRMZM2G023694*, and serine acetyltransferase 1 and 3 of *GRMZM2G069203*-*GRMZM5G816110*. Similar results have been reported in rice and *Arabidopsis* [36]. We previously discovered that the ubiquitin pathway is related to the RBSDV-response pathway in maize [15]. Some FGs with different expressions were also detected, including *GRMZM2G108537*, *GRMZM2G088053*, *AC217499.3_FG003*, *GRMZM2G475380*, *GRMZM2G046331*, and *GRMZM2G404702*, with the following annotation functions, respectively: nodulin protein, WAT1 (walls are thin 1)-related protein, putative peptidase C48 domain family protein, flavone synthase typeI2, a mediator of RNA polymerase II transcription subunit 33A, and cation-transporting ATPase HMA5. The nodulin protein families are involved in plant diseases, such as the colonization of soybean by an arbuscular mycorrhizal fungus [69], the negative regulation of resistance against biotrophic pathogens in *Arabidopsis* [70], and African strains of Xanthomonas oryzae pv. oryzae of rice [71]. The nodulin/glutamine synthetase-like protein (NodGS) is a fusion protein in *Arabidopsis thaliana*, which occurs in several plant genomes. This fusion protein is involved in the regulation of root morphogenesis flagellin-triggered signaling [72,73]. We found that nodulin proteins (*GRMZM2G108537*) are related to RBSDV response in maize. The WAT1-related protein, which is another FG, is likely involved in the gibberellin metabolism of vegetative growth and bast fiber biosynthesis [74]. This indicates that *GRMZM2G088053*, which is a WAT1-related protein in maize, could be correlated with the abortive disease symptoms of MRDD.

### 4.4. Functional Signatures of DEGs, DSGs, and FGs in Maize Response to RBSDV

Three GO properties were enriched in DSG&DEG&FG-overlapped genes: biological processes, cellular components, and molecular functions (Figure 5). During biological processes, GOs come primarily from pathogenesis (GO:0009405), type I hypersensitivity (GO:0016068), apoptotic processes (GO:0006915), the photosystem I reaction center (GO:0009538), response to freezing (GO:0050826), transcription and protein-related processes, ion transport, metabolic processes, and transport. GO:0009405 was significantly weaker during wheat ear infections during the pathogenesis of both F. culmorum and Fusarium graminearum [73]. In our study, nine genes were involved in GO:0009405 response to RBSDV infections in maize, including three DEGs, six DSGs, and two FGs. Of these, *GRMZM2G072612* was recognized with both DEG and DSG, and *GRMZM2G108537* with both DEG and FG. In GO:0009405, six genes were annotated with a downstream target of AGL15 2, including integral membrane protein-like, nodulin protein, nuclear pore complex protein NUP1, protein-enhanced disease resistance 2, and translation factor GUF1 homolog-mitochondrial. Previous studies have found that the nodulin/glutamine synthetase-like fusion protein found in *Arabidopsis* is involved in root morphogenesis regulation, as well as flagellin-triggered signaling [72]. *GRMZM2G108537* is annotated with nodulin proteins, whereas DEGs were found to be related to MRDD in our previous study. In this study, we found that this gene could be involved in gene fusion events to respond to RBSDV infections in maize [15]. *GRMZM2G108537* could be responsible for MRDD symptoms by leaving a waxy white bump on plant leaves.

Hypersensitive response (HR) refers to a cell-death site where the pathogen enters the body and is associated with resistance. HR is associated with programmed cell death (PCD), which is regulated by the dying cell and requires assistance from nearby tissues. The UBC13 in *Arabidopsis*, which results in hypersensitivity, regulates two different cell-death sites that respond to low-temperature stresses and pathogens [75]. In rice, the NH1/OsNPR1 paralog can enhance disease resistance and hypersensitivity and participate in cell death [76]. In maize, *ZmHIR3* is a hypersensitive response-related gene that increases resistance to *Gibberella stalk rot* [77]. We found that hypersensitive and apoptotic processes are likely involved in the response to RBSDV infections in maize. During the GO:0006915//apoptotic process, six DEGs, two DSGs, and one FG responded to RBSDV in maize, including five annotated genes. The CC-NBS-LRR genes (*GRMZM2G032602*, disease resistance protein RPS2 in GO:0006915//apoptotic process) play important roles in the resistance to *Aspergillus flavus* and *Fusarium verticillioides* in maize kernels [78,79]. Three BAG family genes were identified during the GO:0006915//apoptotic process, which is needed for fungal resistance and autophagy in plants [80]. Two BAG family genes were detected during the GO:0006915//apoptotic process and GO:0015979//photosynthesis. Three genes (*GRMZM5G851490*, *GRMZM2G077752*, and *GRMZM2G114893*) were detected during the GO:0006915//apoptotic process and are involved in stem processes, leaf senescence, and resistance to common rust (Puccinia sorghi), respectively, in maize [81,82,83].

During GO:0016068//type I hypersensitivity, three RBSDV-response genes were explored with annotation functions of chloroplasts (DEG-*GRMZM2G021256*, DSG-*GRMZM2G121960*, and FG-*GRMZM2G109561*). Four genes (*GRMZM2G032602*//disease resistance protein RPS2, *GRMZM2G063162*//BAG family molecular chaperone regulator 6, *GRMZM2G097135*//putative IQ calmodulin-binding, BAG domain-containing family protein, and *GRMZM2G079956*//uncharacterized LOC100384516) were recognized in two important GOs: GO:0006915//apoptotic process and GO:0015979//photosynthesis. Previous studies have found that chloroplasts are affected by viral infections [84] and immunity [85]. Our previous study found that healthy B73 chloroplasts developed with a normal ultrastructure, internal matrix, grana, thylakoids, and starch grains. However, once they were infected with RBSDV, the composition of the external and internal structures of the chloroplasts changed [15]. DSG&DEG&FG-overlapped genes were from GO:0015979//photosynthesis (annotation: cellular components) and GO:0009538//photosystem I (annotation: biological processes), which was reported in apples and citrus [86,87]. Meanwhile, three genes (*GRMZM2G016622*, *GRMZM2G017290*, and *GRMZM2G026015*) were found in the two photosystem-related GOs. This indicates that pathogenesis, HR, apoptotic processes, and photosystems could be crosslinked among patterns of DEG, DSG, and FG during the early stages of maize response to RBSDV.

Plant response to viral infections is a complex process because of viral variations (particularly RNA viruses) and regulation of the host plant. Plant genomes should be changed to enhance resistance to viruses, including difference expression, gene modification, and sequence variation, such as SNP, InDel, AS, and FG. In RBSDV-infected maize, some genes resisted the virus through AS and FG. Previous studies have demonstrated that RBSDV populations have been subjected to negative and purifying selection, which could be related to the relationship between the plant and the virus. This study detected correlations between the number of DSGs and FGs and resistance to RBSDV in maize. Other genes with a similar approach could produce resistance to viral mutation responses.

## 5. Conclusions

This study characterized globally AS- and FG-mediated transcriptional regulation, for which the coordinated regulation of DEGs, DSGs, and FGs likely plays important role in maize resistance to RBSDV. These results are valuable for post-transcriptional and transcriptional regulation of maize response to RBSDV stress.

## Figures and Tables

**Table 1:** DEG, DSG, and FG analysis of important GOs.

GO and Annotation	Gene	Gene Annotation Function	DEG	DSG	FG
GO:0009405//pathogenesis	*GRMZM2G033746*	downstream target of AGL15 2		DSG	
	*GRMZM2G071378*	integral membrane protein-like		DSG	
	*GRMZM2G108537*	nodulin protein	DEG		FG
	*GRMZM2G329300*	nuclear pore complex protein NUP1		DSG	
	*GRMZM2G160927*	protein-enhanced disease resistance 2		DSG	
	*GRMZM2G028928*	translation factor GUF1 homolog, mitochondrial	DEG		
	*GRMZM2G048434*	uncharacterized LOC100216974		DSG	
	*GRMZM2G072612*	uncharacterized LOC100277067	DEG	DSG	
	*AC186406.4_FG006*				FG
GO:0006915//apoptotic process	*GRMZM2G018988*	BAG family molecular chaperone regulator 6	DEG		
	*GRMZM2G063162*	BAG family molecular chaperone regulator 6	DEG		
	*GRMZM2G032602*	disease resistance protein RPS2	DEG		
	*GRMZM2G081458*	hypothetical protein ZEAMMB73_*Zm00001d047952*	DEG		
	*GRMZM2G097135*	putative IQ calmodulin-binding and BAG domain-containing family protein	DEG		
	*AC195587.4_FG004*	uncharacterized LOC100276046			FG
	*GRMZM2G017013*	uncharacterized LOC100280475		DSG	
	*GRMZM2G079956*	uncharacterized LOC100384516	DEG	DSG	
GO:0016068//type I hypersensitivity	*GRMZM2G033746*	downstream target of AGL15 2		DSG	
	*GRMZM2G413829*	DUF740 family protein	DEG		
	*GRMZM2G121960*	high chlorophyll fluorescence3		DSG	
	*GRMZM2G063827*	hus1-like protein		DSG	
	*GRMZM2G021256*	photosynthetic NDH subunit of lumenal location 2, chloroplastic	DEG		
	*GRMZM2G127632*	probable serine/threonine-protein kinase SIS8		DSG	FG
	*GRMZM5G851490*	probable WRKY transcription factor 47	DEG		
	*GRMZM2G077752*	protein lateral root primordium 1	DEG		
	*GRMZM2G096169*	putative DUF231 domain-containing family protein		DSG	
	*GRMZM2G163291*	single myb histone 6		DSG	
	*GRMZM2G109561*	threonine dehydratase 1 biosynthetic, chloroplastic			FG
	*GRMZM2G148884*	uclacyanin-2	DEG		
	*GRMZM2G114893*	zinc finger (C2H2 type) family protein	DEG		
	*GRMZM2G071147*	uncharacterized LOC100191399		DSG	
	*GRMZM2G148180*	uncharacterized LOC100192881		DSG	
	*GRMZM2G180328*	uncharacterized LOC100216773	DEG		
	*GRMZM2G449681*	uncharacterized LOC100281160	DEG		
	*GRMZM5G838098*	uncharacterized LOC100281912	DEG	DSG	
	*GRMZM2G464976*	uncharacterized LOC100384168		DSG	
	*GRMZM2G019050*	uncharacterized LOC100384829		DSG	
	*GRMZM2G173419*	uncharacterized LOC103647186	DEG		
	*GRMZM2G321053*	uncharacterized LOC107546779	DEG		
GO:0009538//photosystem I reaction center	*GRMZM2G017290*	photosystem I reaction center subunit III			FG
	*GRMZM2G024150*	photosystem I subunit d1	DEG		
	*GRMZM2G016622*	uncharacterized LOC100283085		DSG	
	*GRMZM2G026015*	uncharacterized LOC100383080		DSG	
GO:0015979//photosynthesis	*GRMZM2G063162*	BAG family molecular chaperone regulator 6	DEG		
	*GRMZM2G032602*	disease resistance protein RPS2	DEG		
	*GRMZM2G081458*	hypothetical protein ZEAMMB73_*Zm00001d047952*	DEG		
	*GRMZM2G017290*	photosystem I reaction center subunit III			FG
	*GRMZM2G174984*	photosystem II3			FG
	*GRMZM2G163809*	prenyl transferase	DEG		
	*GRMZM2G163658*	probable DNA helicase MCM8			FG
	*GRMZM2G097135*	putative IQ calmodulin-binding and BAG domain-containing family protein	DEG		
	*GRMZM2G112728*	Solanesyl diphosphate synthase 2 chloroplastic		DSG	
	*GRMZM2G016622*	uncharacterized LOC100283085		DSG	
	*GRMZM2G026015*	uncharacterized LOC100383080		DSG	
	*GRMZM2G123667*	uncharacterized LOC100384376		DSG	
	*GRMZM2G079956*	uncharacterized LOC100384516	DEG		

## Data Availability

RNA-seq data are available in the database at NCBI SRA database: PRJNA299369; SRR number: from SRR2758150 to SRR2758159, from SRR2758161 to SRR2758170, from SRR2758174 to SRR2758177. The data that support the findings of this study are available from the corresponding author upon reasonable request.

## Supplementary Materials

The following supporting information can be downloaded at: https://www.mdpi.com/article/10.3390/genes13030456/s1, Table S1: Primers used in experimental validation of stress responsive AS and DEGs; Table S2: Introduction of twelve DSGs which contain three or four AS modes; Table S3: Conjoint Analysis of DEGs and DSGs; Table S4: Detailed introduction of fusion gene response to RBSDV in maize; Table S5: The raw data for the real-time PCR.
